# Seroprevalence of Neutralizing Antibodies against Human Adenovirus Type-5 and Chimpanzee Adenovirus Type-68 in Cancer Patients

**DOI:** 10.3389/fimmu.2018.00335

**Published:** 2018-03-07

**Authors:** Hua Zhao, Can Xu, Xiaoli Luo, Feng Wei, Ning Wang, Huiying Shi, Xiubao Ren

**Affiliations:** ^1^Department of Immunology, Tianjin Medical University Cancer Institute & Hospital, Tianjin, China; ^2^National Clinical Research Center for Cancer, Tianjin, China; ^3^Key Laboratory of Cancer Prevention and Therapy, Tianjin, China; ^4^Tianjin’s Clinical Research Center for Cancer, Tianjin, China; ^5^Key Laboratory of Cancer Immunology and Biotherapy, Tianjin, China; ^6^Bioroc Pharmaceutical & Biotech Company, Tianjin, China; ^7^Tianjin Genstar Vaccine Limited Liability Company, Tianjin, China

**Keywords:** chimpanzee adenovirus serotype-68, human adenovirus serotype-5, neutralizing antibodies, cancer vaccine, cancer immunotherapy

## Abstract

Since the preclinical results about chimpanzee adenovirus serotype-68 (AdC68)-based vaccine showed an encouraging results, it reminded us that AdC68 may be a suitable cancer vaccine vector. Previous study indicated that the seroprevalence of neutralizing antibodies (NAbs) against adenovirus was different between cancer patients and healthy volunteers. Knowledge regarding the prevalence rates of AdC68 NAbs for cancer patients is lacking. Therefore, assessing the preexistence of NAbs against AdC68 in cancer patients could provide useful insights for developing future AdC68-based cancer vaccines. In this study, 440 patients with different pathological types of tumors and 204 healthy adult volunteers were enrolled to evaluate the NAbs against AdC68 and human adenovirus serotype-5 (AdHu5). The seroprevalence of NAbs against AdC68 was much lower than that against AdHu5 in cancer subjects (43.64 vs. 67.05%, *P* < 0.01). The seroprevalence rates of NAbs to AdC68 in the cancer subjects were statistically higher than those detected in the healthy adult volunteers (43.64 vs. 23.53%, *P* = 0.000). The seroprevalence rates of AdC68 NAbs were much lower in lung, laryngeal, esophageal, and cervical cancer patients compared with oropharyngeal, colon, and rectal cancer patients. Furthermore, the seroprevalence rates of AdC68 NAbs were much lower in lung adenocarcinoma patients than in lung squamous cell carcinoma patients (35.00 vs. 70.00%, *P* < 0.05). No significant difference in the AdC68 NAbs among patients with different clinical stages of cancer was detected. The percentage of NAbs against AdC68 was significantly lower than that against AdHu5 (*P* < 0.05) in stage-I, -II, and -III cancer patients. No significant difference between the percentage of NAbs against AdC68 and AdHu5 in the subjects with stage-IV cancer was detected. The study also demonstrated the distribution of AdHu5 and AdC68 NAb titers for the positive samples. It showed that very low NAb titers against AdC68 with respect to AdHu5 in both healthy subjects and cancer subjects, especially in lung, laryngeal, esophageal, gastric, and cervical carcinomas. Also, the titer of NAbs against AdC68 was significantly lower than that against AdHu5 in the same clinical stage and age group (*P* < 0.05). Taken together, the present study showed that NAbs against AdC68 is much lower than AdHu5, especially in lung adenocarcinoma, laryngeal cancer, esophageal cancer, and cervical cancer patients. These results provided strong support for candidating AdC68 as a suitable vector of cancer vaccines.

## Introduction

Therapeutic cancer vaccines based on replication-defective adenoviral vectors have been developing with varying levels of success. Functional tumor antigen-specific CD8^+^ T cells should be activated to achieve tumor regression (1, 2). Adenoviral vectors derived from the common human adenovirus serotype-5 (AdHu5) vector are considered the most well-studied vector because of their ability to induce potent transgene product-specific T- and B-cell responses in experimental animals (3–5) and clinical trials as carriers for HIV-1 vaccines (6). However, the neutralizing antibodies (NAbs) to AdHu5 virus are common in humans and therefore the immune response of the transgene products is often dampened (7–9). To circumvent the problem of preexisting immunity, nonhuman adenovirus vectors, which isolated from chimpanzees with NAbs of lower seroprevalence than that of AdHu5, have been well characterized and developed as vaccine carriers (10–13).

The chimpanzee adenovirus serotype-68 (AdC68) has been shown to be a good gene carrier in vaccine development owing to its high transduction efficiency, broad cell tropism, high gene expression, good genetic stability, and low seropositive rate in humans (13, 14). It can grow in HEK293 cells as sufficient AS and exhibits no significant cross-neutralization by sera against human serotypes including AdHu2, 4, 5, 7, and 12 (10). Like AdHu5, previous study demonstrated thatAdC7- and AdC68-based vaccines could also induce strong T- and B-cell responses to exogenous antigens in mice and nonhuman primates (15). Various vaccine candidates based on AdC68 have been developed for controlling different infectious diseases, including influenza of H5N1 and H7N9 (16, 17). Preclinical study has also demonstrated that the hexon-modified AdC68-based vaccine can induce immune protection against EV71 and CA16 challenge in mice (18). An AdC68-based rabies virus vaccine, termed AdC68rab.gp, induced sustained central and mucosal antibody responses to rabies virus and provides complete protection against rabies virus challenge (19). Early study also reported that AdHu5 vector-based vaccine induced a potent transgene product-specific CD8 + T-cell response that can be increased substantially by using AdC68 vector-based vaccine expressing the same transgene product for booster immunization. This type of heterologous prime-boost regimen is far more effective than priming with a DNA vaccine followed by booster immunization with an Ad recombinant or by using the same Ad vaccine carrier repeatedly (20). The AdC68-based vaccine could also induce protective effector and memory T-cell responses against malignant melanoma cells in mice (21).

As we know, there were no clinical trials based on AdC68 vectors. Since the preclinical results about AdC68-based vaccine showed an encouraging result, it reminded us that AdC68 may be a suitable cancer vaccine vector. However, previous study indicated that the seroprevalence of NAbs against adenovirus was different between cancer patients and healthy volunteers (22). The studies comparing the prevalence of NAbs specific for AdHu5 and AdC68 have mainly been limited to healthy human subjects (13, 20). Knowledge regarding the prevalence rates of AdC68 NAbs for cancer patients is lacking. Assessing the preexistence of NAbs against AdC68 in cancer patients could provide useful insights for developing future AdC68-based cancer vaccines.

## Materials and Methods

### Human Samples

Human serum and plasma samples were obtained from 204 healthy adult volunteers and 440 cancer patients from Tianjin Cancer Hospital, including eight types of cancer patients: 80 lung cancer patients, 35 laryngeal cancer patients, 9 oropharyngeal cancer patients, 76 esophageal cancer patients, 40 gastric cancer patients, 120 cervical cancer patients, 40 colon cancer patients, and 40 rectal cancer patients. All patients enrolled in this study were pathologically diagnosed according to the National Comprehensive Cancer Network Clinical Practice Guidelines. All serum samples were stored at −80°C prior to use, and sera/plasma were heat inactivated at 56°C for 30 min.

### Recombinant Adenoviruses

Replication-defective, green fluorescent protein (GFP)-expressing adenovirus vectors derived from serotypes AdHu5 and AdC68 were purchased from Jikai (Shanghai, China) and Bioroc (Tianjin, China), respectively.

### Adenovirus Neutralization Assay

Adenovirus neutralization assays were performed as described previously (20). Briefly, heat-inactivated sera were added to 96-well plates after serial doubling dilutions (1:10–1:1,280) were mixed with 1 × 10^7^ AdHu5 and AdC68 virus particles expressing GFP and incubated for 1 h at 37°C in a 5% CO_2_ atmosphere. Dulbecco minimum essential medium (DMEM) was used as the negative control. Serum from BALB/c mice primed and boosted *via* the intramuscular route with 1 × 10^11^ GFP-expressing AdHu5 and AdC68 viral particles were used as positive controls. In total, 2.5 × 10^4^ HEK293 cells per well were added to the 96-well plate and mixed fully with the medium. The results were read after 24 h of incubation. The NAb titers are expressed as the reciprocal of dilution in which the ratio of GFP-expressing cells was reduced to approximately 50% compared with that of the negative control. Titers >20 were scored as positive for the presence of serotype-specific NAbs.

### Statistical Analysis

Statistical Package for the Social Sciences version 21.0 (IBM Corp., USA) was applied to perform all the statistical analysis. The chi-square test was used to compare the seroprevalence rates of AdHu5 and AdC68. The Friedman ANOVA test was used to compare the NAb titers among the adenoviral serotypes. In all the tests, the values of *P* < 0.05 using two-sided tests were considered statistically significant.

## Results

### Study Participants and Characteristics

The characteristics of the cancer patient and healthy adult volunteer participants are detailed in Table 1. Sex and age were comparable between the cancer patients and healthy adult volunteers.

**Table 1:** Demographics of study participants with assayed samples (*N* = 644).

	Cancer patients (*n*, %)	Healthy adult volunteers (*n*, %)
**Age groups (years)**		
<60	258 (58.64%)	112 (54.90%)
≥60	182(41.36%)	92 (45.10%)
**Gender**		
Male	228 (51.82%)	108 (52.94%)
Female	212 (48.18%)	96 (47.06%)
**Pathological type**		
Squamous cell carcinoma	280 (63.64%)	
Adenocarcinoma	160 (36.36%)	
**Clinical stage**		
Stage I	133 (30.23%)	
Stage II	162 (36.82%)	
Stage III	115 (26.14%)	
Stage IV	30 (6.82%)	

### Seroprevalence of Anti-Adenovirus NAbs

In this study, 204 samples from healthy adult volunteers and 440 samples from cancer patients were evaluated for their NAbs against AdHu5 and AdC68. The seroprevalence rates in the total samples of NAbs against AdHu5 were statistically higher than that of AdC68 (68.48 vs. 37.27%, *P* < 0.01, Table 2). In the group of healthy adult volunteers, the seroprevalence rates of NAbs against AdHu5 were statistically higher than that of AdC68 (71.57 vs. 23.53%, *P* < 0.01). In the group of cancer patients, the seroprevalence rates of NAbs against AdHu5 were statistically higher than that of AdC68 (67.05 vs. 43.64%, *P* < 0.01). Taken together, these results indicate that preexisting immunity is more common against AdHu5 than against AdC68, suggesting the use of AdC68 vectors rather than AdHu5 vectors as vaccine carriers.

**Table 2:** Seroprevalence of AdHu5 and AdC68 neutralizing antibody in cancer patients and healthy adult volunteers.

	AdHu5	AdC68	*P-*value
Total samples	68.48% (441/644)	37.27% (240/644)	0.000
Healthy adult Volunteers	71.57% (146/204)	23.53% (48/204)	0.000
Cancer patients	67.05% (295/440)	43.64% (192/440)	0.000

No significant difference in the seroprevalence rates of NAbs against AdHu5 between healthy adult volunteers and cancer patients existed (*P* > 0.05). However, the seroprevalence rates of NAbs against AdC68 in the cancer patient samples were statistically higher than that of healthy adult volunteers (43.64 vs. 23.53%, *P* = 0.000). The results indicated that preexisting immunity against AdC68 is more common in cancer patients than in healthy volunteers.

**Figure 1:**
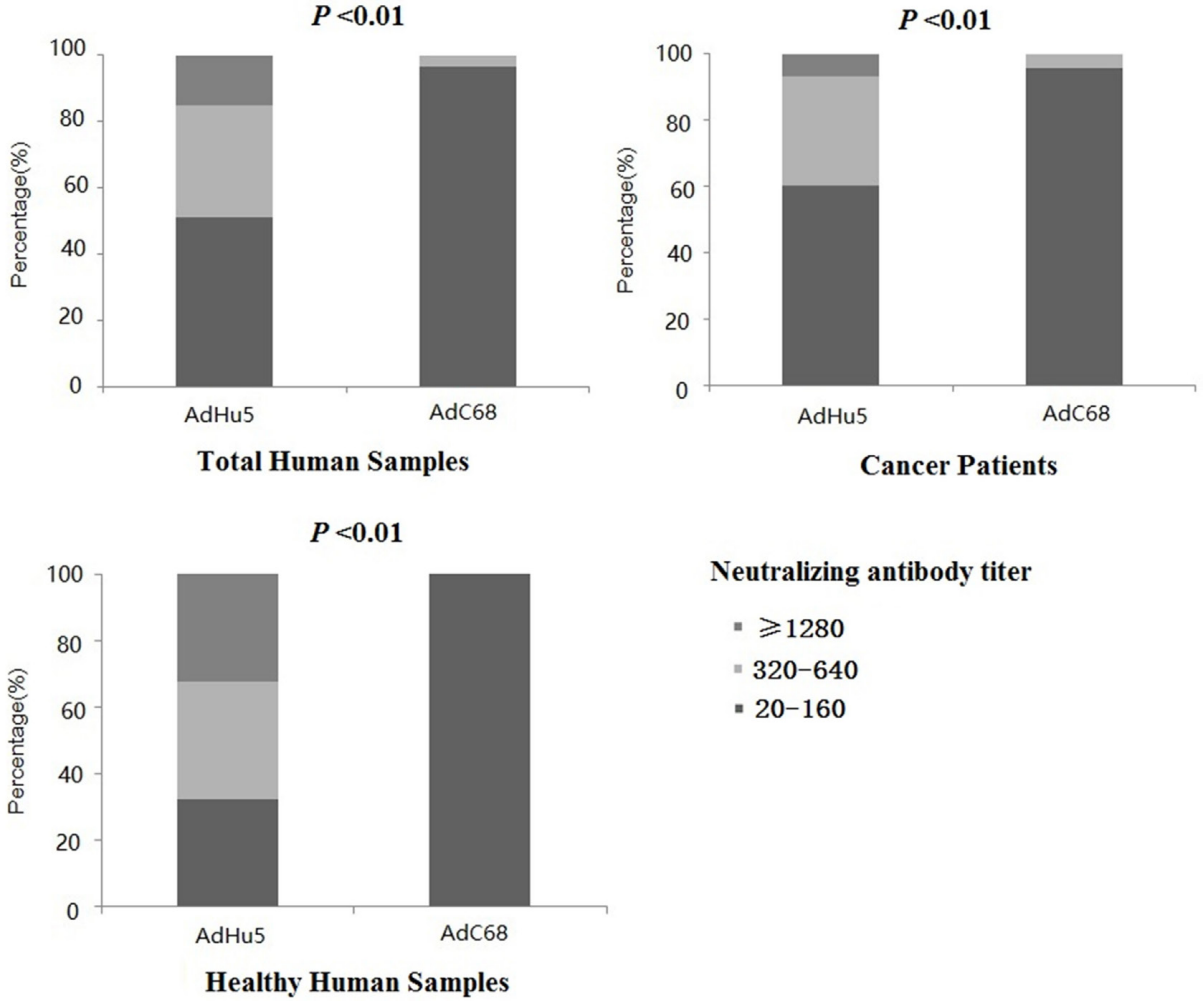
Distribution of neutralizing antibody (NAb) titers against AdHu5 and AdC68 in positive samples. NAb titers were stratified in the following tiers: low titer (≥20 and ≤160), medium titer (≥320 and ≤640), and high titer (≥1,280). Across positive samples from all of the human subjects, the NAb titers against AdHu5 from low to high were 51.02, 33.79, and 15.19%, respectively. The AdC68 NAb titers in positive samples from low to high were 96.67, 3.33, and 0%, respectively, which was significantly different from AdHu5 (*P* < 0.01). For positive samples from the cancer subjects, the NAb titers against AdHu5 from low to high were 51.02, 33.79, and 15.19%, respectively. However, most cancer subjects exhibited low NAb titers against AdC68 (≥20 and ≤160, 95.83%), and a small fraction of cancer subjects exhibited a medium level of AdC68 NAb titers (≥320 and ≤640, 4.17%). For the healthy adult volunteers, the AdHu5 NAb titers in the positive samples from low to high were 32.19, 35.62, and 32.19%, respectively, whereas all of titers from the positive NAb samples against AdC68 were lower or equal to 160.

In this study, we further analyzed the distribution of AdHu5 and AdC68 NAb titers for the positive samples (Figure 1, detailed data showed in Table S1 in Supplementary Material). The NAb titers were stratified across the following tiers: low titers (≥20 and ≤160), medium titers (≥320 and ≤640), and high titers (≥1,280). For all of the human subjects, the AdHu5 NAb titers for positive samples from low to high titers were 51.02, 33.79, and 15.19%, respectively. In contrast, the AdC68 NAb titers for positive samples from low to high titers were 96.67, 3.33, and 0%, respectively. For the healthy adult volunteers, the AdHu5 NAb titers for positive samples from low to high titers were 32.19, 35.62, and 32.19%, respectively, whereas all of the positive AdC68 NAb sample titers were lower or equal to 160. Most cancer patient subjects exhibited low AdHu5 NAb titers (≥20 and ≤160, 60.34%), and a small fraction exhibited very high AdHu5 NAb titers (≥1,280, 6.78%). In contrast to this distribution, most cancer patient subjects exhibited low NAb titers against AdC68 (≥20 and ≤60, 95.83%), and a small fraction of cancer patients exhibit a medium level of AdC68 NAb titers (≥320 and ≤640, 4.17%).

### Seroprevalence of Anti-Adenovirus NAbs in Different Cancer Types

Subjects with eight different types of cancer were evaluated for their levels of AdHu5 and AdC68 NAbs (Table 3). The percentages of NAbs against AdC68 were significantly lower in lung, laryngeal, esophageal, and cervical carcinoma patients than those against AdHu5 (*P* < 0.05). No significant differences between the percentages of NAbs against AdC68 and AdHu5 in oropharyngeal, gastric, rectal, or colon cancer patients were detected. These results indicate that preexisting immunity against AdC68 is rare in laryngeal, esophageal, and cervical carcinomas, which suggests that AdC68 vectors may be considered vaccine carriers in these types of cancers.

**Table 3:** Seroprevalence of AdHu5 and AdC68 neutralizing antibody in patients with different types of cancer.

	AdHu5	AdC68	*P-*value
Lung cancer	75.00% (60/80)	52.50% (42/80)	0.003
Laryngeal cancer	88.57% (31/35)	25.71% (9/35)	0.000
Oropharyngeal cancer	66.67% (6/9)	66.67% (6/9)	1.000
Esophageal cancer	67.11% (51/76)	32.89% (25/76)	0.000
Gastric cancer	77.50% (31/40)	75.00% (30/40)	0.793
Cervical carcinoma	54.17% (65/120)	26.67% (32/120)	0.000
Rectal cancer	65.00% (26/40)	70.00% (28/40)	0.633
Colon cancer	62.50% (25/40)	50.00% (20/40)	0.260

We also analyzed the distribution of AdHu5 and AdC68 NAb titers for positive samples in the different types of cancer subjects (Table 4). For the subjects with lung, laryngeal, esophageal, gastric, and cervical carcinomas, the distributions of AdHu5 NAb titers for positive samples were different from the distribution of AdC68 NAb titers (*P* < 0.05). No differences in the distributions between the AdHu5 and AdC68 NAb titers for positive subjects with oropharyngeal, rectal, or colon cancer were detected.

**Table 4:** Distribution of AdHu5 and AdC68 neutralizing antibody titers in positive samples from subjects from different type of cancer.

	High (20–160 titers)	Medium (320–640 titers)	Low (≥1,280 titers)	*P*-value
Lung cancer				0.000
AdHu5 neutralizing antibody	37 (61.67%)	18 (30.00%)	5 (8.33%)	
AdC68 neutralizing antibody	40 (95.24%)	2 (4.76%)	0	
Laryngeal cancer				0.005
AdHu5 neutralizing antibody	14 (45.16%)	17 (54.84%)	0	
AdC68 neutralizing antibody	9 (100.0%)	0	0	
Oropharyngeal cancer				0.301
AdHu5 neutralizing antibody	4 (66.66%)	1 (16.67%)	1 (16.67%)	
AdC68 neutralizing antibody	6 (100.0%)	0	0	
Esophageal cancer				0.028
AdHu5 neutralizing antibody	22 (42.31%)	23 (44.23%)	7 (13.46%)	
AdC68 neutralizing antibody	6 (100.0%)	0	0	
Gastric cancer				0.011
AdHu5 neutralizing antibody	25 (80.65%)	6 (19.35%)	0	
AdC68 neutralizing antibody	30 (100.0%)	0	0	
Cervical carcinoma				0.001
AdHu5 neutralizing antibody	39 (60.0%)	20 (30.77%)	6 (9.23%)	
AdC68 neutralizing antibody	31 (96.88%)	1 (3.12%)	0	
Rectal cancer				0.496
AdHu5 neutralizing antibody	20 (76.92%)	5 (19.23%)	1 (3.85%)	
AdC68 neutralizing antibody	24 (85.71%)	4 (14.29%)	0	
Colon cancer				0.077
AdHu5 neutralizing antibody	17 (68.0%)	7 (28.0%)	1 (4.0%)	
AdC68 neutralizing antibody	19 (95.0%)	1 (5.0%)	0	

### Seroprevalence of Anti-Adenovirus NAbs in Cancer Patients of Different Clinical Stages

We further analyzed the seroprevalence of AdHu5 and AdC68 NAbs in cancer patients in different clinical stages of their diseases (Table 5). No significant difference in the AdC68 NAbs was detected among patients of different disease stages (*P* = 0.849). The percentages of NAbs against AdC68 were significantly lower than those against AdHu5 (*P* < 0.05) in subjects of stages I, II, and III. No significant difference in the percentages of NAbs against AdC68 and AdHu5 in subjects of stage IV was detected.

**Table 5:** Seroprevalence of AdHu5 and AdC68 neutralizing antibodies in subjects with different clinical stage of cancer.

Clinical stage	AdHu5	AdC68	*P-*value
Stage I	61.65% (82/133)	41.35% (55/133)	0.001
Stage II	66.67% (108/162)	43.83% (71/162)	0.000
Stage III	72.17% (83/115)	44.35% (51/115)	0.000
Stage IV	73.33% (22/30)	50.00% (15/30)	0.063

**Figure 2:**
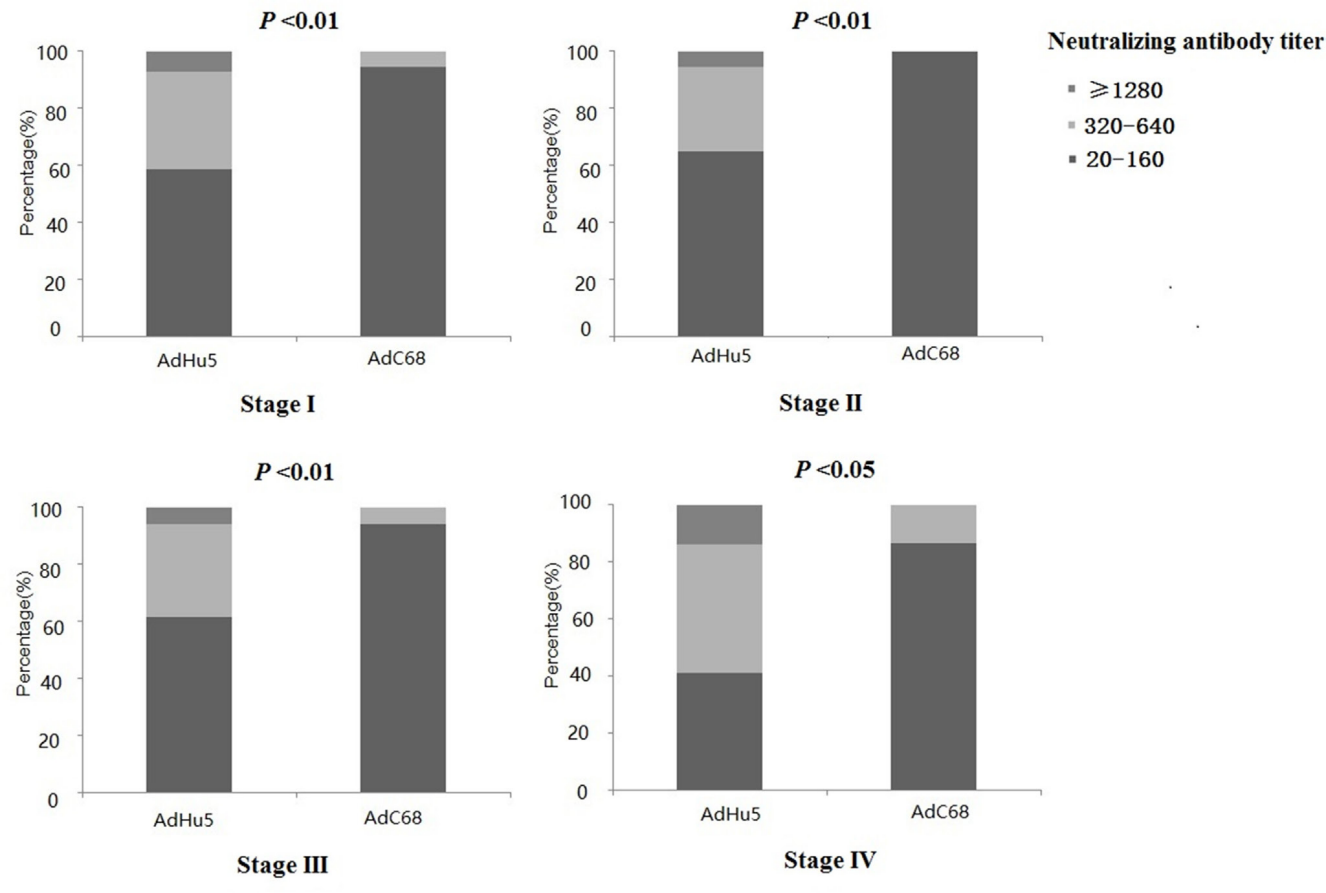
Distribution of AdHu5 and AdC68 neutralizing antibody (NAb) titers in positive samples from cancer patients in different clinical stages. NAb titers were stratified in the following tiers: low titer (≥20 and ≤160), medium titer (≥320 and ≤640), and high titer (≥1,280). For stage-I positive subjects, the AdHu5 NAb titers from low to high were 58.54, 34.15, and 7.32%, respectively, whereas the AdC68 NAb titers from low to high were 94.55, 5.45, and 0%, respectively; a significant difference existed between the AdHu5 NAb and AdC68 NAb titers among stage-I positive subjects (*P* < 0.01). For stage-II positive subjects, the AdHu5 NAb titers from low to high were 64.81, 29.63, and 5.56%, respectively, while all of the positive AdC68 NAb sample titers were lower or equal to 160. For stage-III positive subjects, the AdHu5 NAb titers from low to high were 61.45, 32.53, and 6.02%, respectively. However, most cancer patients exhibited low NAb titers against AdC68 (≥20 and ≤160, 94.12%), and a small fraction of cancer patients exhibited a medium level of AdC68 NAb titers (≥320 and ≤640, 5.88%). For stage-IV positive subjects, the AdHu5 NAb titers from low to high were 40.91, 45.45, and 13.64%, respectively, while the AdC68 NAb titers from low to high were 86.67, 13.33, and 0%, respectively; this was significantly different from AdHu5 (*P* < 0.05).

We also analyzed the distribution of AdHu5 and AdC68 titers for positive samples in different clinical stages, and a significant difference between these two vectors was detected (*P* < 0.05, Figure 2, detailed data showed in Table S1 in Supplementary Material). For stage-I positive subjects, the AdHu5 NAb titers from low to high were 58.54, 34.15, and 7.32%, respectively, while the AdC68 NAb titers from low to high were 94.55, 5.45, and 0%, respectively. A significant difference between AdHu5 NAb and AdC68 Nab titers was detected in stage-I positive subjects (*P* < 0.01). For stage-II positive subjects, the AdHu5 NAb titers from low to high were 64.81, 29.63, and 5.56%, respectively, while all of the positive AdC68 NAb sample titers were lower than or equal to 160, which was significantly different from AdHu5 (*P* < 0.01). For stage-III positive subjects, the AdHu5 NAb titers from low to high were 61.45, 32.53, and 6.02%, respectively. However, most cancer patient subjects exhibited low NAb titers against AdC68 (≥20 and ≤160, 94.12%) and a small fraction of cancer patients exhibited a medium level of AdC68 NAb titers (≥320 and ≤640, 5.88%). The distribution of these two vectors in stage-III patients was significantly different (*P* < 0.01). For stage-IV positive subjects, the AdHu5 NAb titers from low to high were 40.91, 45.45, and 13.64%, respectively. However, the AdC68 NAb titers from low to high were 86.67, 13.33, and 0%, respectively, which was significantly different than that of AdHu5 (*P* < 0.05).

### Seroprevalence of Anti-Adenovirus NAbs in Subjects with Different Pathological Types of Cancer

In this study, we also analyzed the seroprevalence of anti-adenovirus NAbs in subjects with different pathological types of cancer. Because the laryngeal, oropharyngeal, esophageal, and cervical cancers were all squamous cell carcinomas, the seroprevalence rates of AdHu5 and AdC68 NAbs in squamous cell carcinomas could be shown together in Table 2. Because the gastric, rectal, and colon cancers were all adenocarcinomas, such as the squamous cell carcinomas, the seroprevalence of AdHu5 and AdC68 NAbs in adenocarcinomas could be the same within these three cancer subjects. Therefore, we analyzed the seroprevalence of AdHu5 and AdC68 NAbs in different pathological types of lung cancer.

The seroprevalence of AdHu5 NAbs in lung adenocarcinoma subjects was significantly higher than that of AdC68 (77.50 vs. 35.00%, *P* < 0.01 Table 6). In contrast, no difference between the seroprevalence of AdHu5 and AdC68 in the lung squamous cell carcinoma subjects was detected (72.50 vs. 70.00%, *P* > 0.05). No significant difference in the seroprevalence rates of NAbs against AdHu5 between the lung adenocarcinoma and lung squamous cell carcinoma subjects was detected (77.50 vs. 72.50%, *P* = 0.606). However, the seroprevalence rates of NAbs against AdC68 in the samples from lung squamous cell carcinoma subjects were statistically higher than those from lung adenocarcinoma patients (70.00 vs. 35.00%, *P* = 0.049).

**Table 6:** Seroprevalence of AdHu5 and AdC68 neutralizing antibody in subjects with different pathological type of lung cancer.

	AdHu5	AdC68	*P-*value
Squamous cell carcinoma	72.50% (29/40)	70.00% (28/40)	0.805
Adenocarcinoma	77.50% (31/40)	35.00% (14/40)	0.008

**Figure 3:**
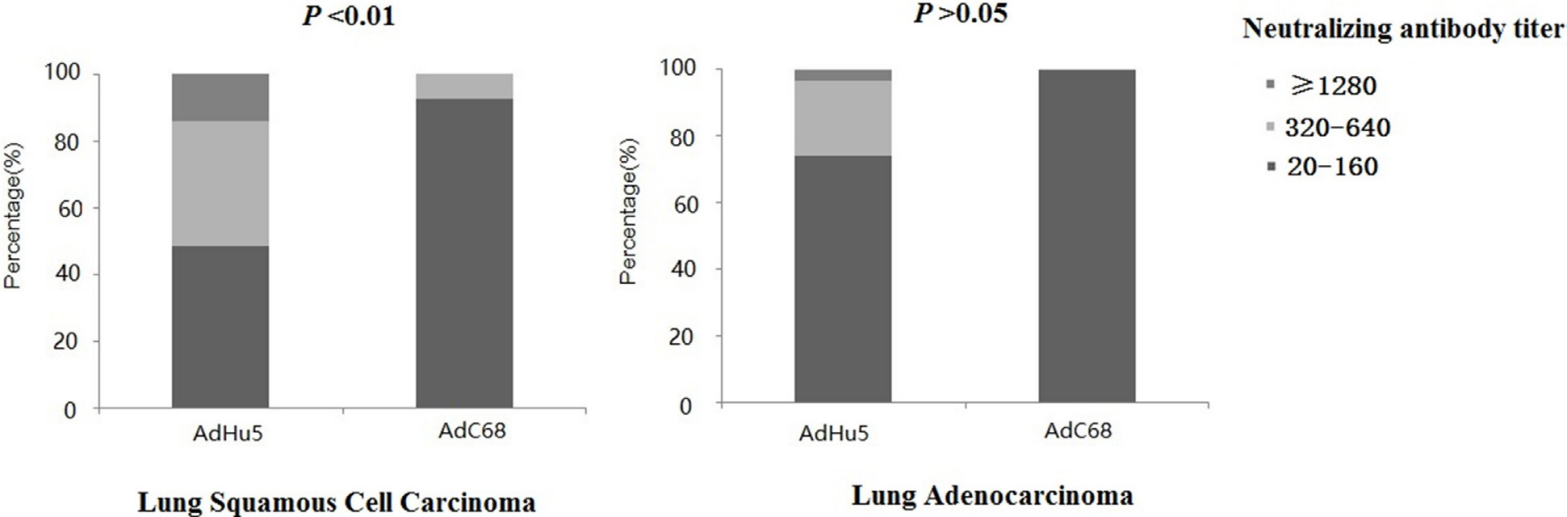
Distribution of AdHu5 and AdC68 neutralizing antibody (NAb) titers in positive samples from lung squamous cell carcinoma and adenocarcinoma subjects. NAb titers were stratified in the following tiers: low titer (≥20 and ≤160), medium titer (≥320 and ≤640), and high titer (≥1,280). For the positive samples from lung squamous cell carcinoma patients, the AdHu5 NAb titers from low to high were 48.28, 37.93, and 13.79%, respectively, while the AdC68 NAb titers from low to high were 92.86, 7.14, and 0%, respectively; this was significantly different from AdHu5 (*P* < 0.05). For positive samples from lung adenocarcinoma patients, the AdHu5 NAb titers from low to high were 74.19, 22.58, and 3.23%, respectively, while all of the positive AdC68 NAb sample titers were lower or equal to 160. However, no significant difference in the distribution of the two vectors in lung adenocarcinoma patients was observed (*P* > 0.05).

We further analyzed the distribution of AdHu5 and AdC68 NAb titers among the positive lung cancer subjects (Figure 3). For the positive lung squamous cell carcinoma samples, the AdHu5 NAb titers from low to high were 48.28, 37.93, and 13.79%, respectively, while the AdC68 NAb titers from positive samples from low to high were 92.86, 7.14, and 0%, respectively, which was significantly different from AdHu5 (*P* < 0.05). For the positive lung adenocarcinoma samples, the AdHu5 NAb titers from low to high were 74.19, 22.58, and 3.23%, respectively, while all of the positive AdC68 NAb sample titers were lower or equal to 160. However, no significant difference between the distributions of the two vectors was detected in lung adenocarcinoma subjects (*P* > 0.05).

### Seroprevalence of Anti-Adenovirus NAbs in Different Age Groups

In this study, we randomly collected peripheral plasma of 440 cancer patients so that the subjects were not evenly distributed according to the age groups. The subjects were divided into four groups according to their age: 18–39 years of group, 40–49 years of group, 50–59 years of group, and no less than 60 years of group. The seroprevalence rates of NAbs against AdHu5 in the total samples and cancer samples were significantly increased with the increasing age (*P* = 0.038 and 0.005, respectively), so as to the AdC68 (*P* = 0.031 and 0.006, respectively). However, the seroprevalence rates of NAbs against AdHu5 and AdC68 showed no significant difference in the healthy adult volunteers (*P* > 0.05). The seroprevalence rates of NAbs against AdC68 were much higher than AdHu5 in the same age groups except for cancer patients of 18–39 years of group (*P* < 0.05, Table 7). Furthermore, there were no significant differences in the distribution of AdHu5 and AdC68 NAb titers for positive samples in the different age groups of neither the total subjects, healthy subjects, nor cancer subjects (*P* > 0.05, Table 7).

**Table 7:** Seroprevalence of AdHu5 and AdC68 neutralizing antibody in different age groups.

Age groups (years)	*N*	AdHu5 (%)	AdC68 (%)	*P*-value
**Total samples**				
18–39	36	20 (55.56)	9 (25.00)	0.008
40–49	113	69 (61.06)	32 (28.32)	0.000
50–59	218	152 (69.72)	84 (38.53)	0.000
≥60	274	200 (72.99)	115 (41.97)	0.000
**Healthy adult volunteers**				
18–39	11	8 (72.73)	1 (9.09)	0.002
40–49	27	18 (66.67)	7 (25.93)	0.003
50–59	74	58 (78.38)	16 (21.62)	0.000
≥60	92	62 (67.39)	24 (26.09)	0.000
**Cancer patients**				
18–39	25	12 (48.00)	8 (32.00)	0.248
40–49	86	51 (59.30)	25 (29.07)	0.000
50–59	144	94 (65.28)	68 (47.22)	0.002
≥60	182	138 (75.82)	91 (50.00)	0.000

In addition, the titer of NAbs against AdC68 was significantly lower than that against AdHu5 in the same age groups (*P* < 0.05, Table 8). However, there were no significant differences of the titer of NAbs against AdHu5 and AdC68 in different age groups (*P* > 0.05, Table 8).

**Table 8:** Titers of the AdHu5 and AdC68 neutralizing antibody in different age groups.

Age groups (years)	*N*	AdHu5 (%)medium	AdC68 (%)medium	*P*-value
**Total samples**				
18–39	36	20	0	0.001
40–49	113	80	0	0.000
50–59	218	80	10	0.000
≥60	274	80	10	0.000
**Healthy adult volunteers**				
18–39	11	160	0	0.007
40–49	27	160	0	0.000
50–59	74	320	0	0.000
≥60	92	160	0	0.000
**Cancer patients**				
18–39	25	10	0	0.026
40–49	86	40	0	0.000
50–59	144	80	10	0.000
≥60	182	80	20	0.000

## Discussion

Prior studies reported that AdC68 NAbs occur at a lower prevalence in healthy volunteers from North America (2–4%), Southeast Asia (1.5–3%), Africa (2–20%), and China (9.4–21.8%) (23–25), which are much lower than the NAbs to AdHu5 seroprevalence rates (60–100%) in the same regions (26–30). In the present study, AdHu5 NAb prevalence of 68.48% among all the human subjects (*n* = 644) was similar to the positive rate(73.1%) of anti-AdHu5 NAbs observed in 1,154 healthy adults from six regions in China (13). No significant difference in the seroprevalence rates of NAbs to AdHu5 between healthy adult volunteers and cancer patients was observed (71.57 vs. 67.05%, *P* > 0.05). However, the seroprevalence rate for AdC68 NAbs was 37.27% among all the human subjects, which was much lower than that for AdHu5 NAbs. The seroprevalence rates of NAbs against AdC68 in the cancer patient samples were statistically higher than that of healthy adult volunteers (43.64 vs. 23.53%, *P* = 0.000). Similar results were reported in an early study which showed that seroprevalence rate of NAbs against AdC6 and AdC7 was higher in primary hepatocellular carcinoma than that in healthy volunteers (22). Although there is no clear explanation for this phenomenon, cross-reactivity of various viral infections may be involved because of the lower titer distribution of NAbs to AdC68.

A total of 440 cancer subjects were enrolled in this study, which included eight types of cancer. Compared with subjects with other types of cancer, the seroprevalence rates of AdC68 NAbs were much lower in laryngeal (25.71%), esophageal (32.89%), and cervical (26.67%) cancer subjects, which encourages the clinical use of AdC68-based vectors as antitumor vaccine carriers. Because few subjects with oropharyngeal, colon, and rectal cancer were enrolled in this study, the distribution difference of AdC68 NAbs among the different cancer types should be studied with much more samples.

Furthermore, we analyzed the seroprevalence of AdC68 NAbs in different clinical stages of cancer subjects. The seroprevalence rates of AdC68 NAbs were significantly higher than AdHu5 NAbs in stages I, II, and III. Although the seroprevalence rate of AdC68 NAbs was much lower than that of AdHu5 NAbs in stage-IV subjects, there was no significant difference between them. This may be due to the small number of subjects from stage IV.

Interestingly, the seroprevalence rates of NAbs to AdC68 in the lung squamous cell carcinoma samples were statistically higher than those in the lung adenocarcinoma samples (70.00 vs. 35.00%, *P* = 0.049). These results indicated that the AdC68 vector is more suitable as a vaccine carrier for lung adenocarcinoma than that for lung squamous cell carcinoma.

Previous studies showed that NAbs against AdHu5 were commonly present at high titers in human sera, while the NAb titers against AdC68 were much lower (31). High-titer NAbs against adenovirus could affect the immunogenicity and reduce the effectiveness of the vaccine *in vivo* (32–35). Zhang et al. reported that 59.2% of Chinese subjects exhibited AdHu5 NAb titers >160, and 21.2% of the subjects exhibited titers >1,000. They also showed that 2.0% of Chinese subjects exhibited AdC68 NAb titers >160, and 0.0% exhibited titers >1,000 (13). In our study, 32.88% of the cancer subjects exhibited AdHu5 NAb titers >160, and 6.78% exhibited AdHu5 NAb titers >1,000. In total, 4.17% of the cancer subjects harbored AdC68 NAb titers >160, and 0.0% harbored AdC68 NAb titers >1,000. Taken together, we concluded that the AdC68 NAb titers from the positive samples were much lower than the AdHu5 NAb titers.

In addition, we assessed the seroprevalence of NAbs against AdHu5 and AdC68 in different age groups. It showed similar results with the previous studies (13). The titer of NAbs against AdC68 was significantly lower than AdHu5 in the same age groups. However, there were no significant differences with the titer of NAbs against AdHu5 and AdC68 in different age groups.

In conclusion, the seroprevalence rates of NAbs against AdC68 were much lower than those against AdHu5 in cancer subjects, especially in lung adenocarcinomas, laryngeal, esophageal, and cervical cancers. These results could provide useful insights for developing future AdC68-based cancer vaccines. Furthermore, before the clinical trial, the biological distribution of carrier as well as acute and chronic toxic reactions should be evaluated in mice and non-human primates. The prime-boost program should also be evaluated to strength the immune effect.

## Ethics Statement

This study was approved by the Ethical Committee of TMUCIH. Written informed consent was obtained from each subject in accordance with the Declaration of Helsinki.

## Author Contributions

HZ and HS designed the study protocol. HZ wrote this manuscript. HZ, CX, and XL performed the experiments. FW and HZ analyzed the collected data. XR revised the manuscript. All authors read and approved the final manuscript.

## Conflict of Interest Statement

Tianjin Bioroc Pharmaceutical & Biotech Company is mainly engaged in research and development of cancer immunotherapy and related products. Tianjin Genstar Vaccine Limited Liability Company is mainly engaged in research and development of viral vector-based therapeutic tumor vaccines. Tianjin Genstar Vaccine Limited Liability Company is the subsidiary holding by Tianjin Bioroc Pharmaceutical & Biotech Company. CX, XL, NW, and HS are employees of Tianjin Genstar Vaccine Limited Liability Company. HZ, FW, and XR are scientific researchers of Tianjin Medical University Cancer Institute & Hospital and declare that they have no competing interests.
