# Supplementary material for: Seroprevalence of Neutralizing Antibodies against Human Adenovirus Type-5 and Chimpanzee Adenovirus Type-68 in Cancer Patients
Source: Front Immunol. 2018 Mar 7;9:335. doi: 10.3389/fimmu.2018.00335 (PMC5845880; doi:10.3389/fimmu.2018.00335)
Supplement: Supplementary file 1 [file table_1.PDF]

Supplement 1. The actual data of NAb titers against AdHu5 and AdC68 in healthy adult volunteers and cancer patients

Subjects	Nab titer to AdHu5	Nab titer to AdC68
healthy volunteers	20	0
healthy volunteers	160	0
healthy volunteers	10	0
healthy volunteers	40	0
healthy volunteers	160	0
healthy volunteers	20	20
healthy volunteers	10	0
healthy volunteers	10	10
healthy volunteers	10	10
healthy volunteers	160	0
healthy volunteers	10	0
healthy volunteers	10	0
healthy volunteers	10	0
healthy volunteers	160	0
healthy volunteers	160	0
healthy volunteers	640	0
healthy volunteers	80	20
healthy volunteers	80	80
healthy volunteers	10	80

healthy volunteers	2560	160
healthy volunteers	10	0
healthy volunteers	2560	0
healthy volunteers	10	0
healthy volunteers	10	20
healthy volunteers	80	0
healthy volunteers	80	0
healthy volunteers	10	0
healthy volunteers	10	80
healthy volunteers	320	40
healthy volunteers	10	0
healthy volunteers	10	40
healthy volunteers	320	0
healthy volunteers	10	0
healthy volunteers	320	0
healthy volunteers	80	80
healthy volunteers	80	0
healthy volunteers	2560	0
healthy volunteers	80	80
healthy volunteers	2560	0
healthy volunteers	1280	0
healthy volunteers	320	0

healthy volunteers	640	0
healthy volunteers	10	0
healthy volunteers	10	40
healthy volunteers	10	0
healthy volunteers	1280	0
healthy volunteers	640	0
healthy volunteers	20	0
healthy volunteers	1280	0
healthy volunteers	5120	0
healthy volunteers	20	40
healthy volunteers	640	0
healthy volunteers	10	0
healthy volunteers	2560	0
healthy volunteers	2560	0
healthy volunteers	1280	80
healthy volunteers	1280	0
healthy volunteers	640	40
healthy volunteers	10	0
healthy volunteers	0	0
healthy volunteers	320	0
healthy volunteers	1280	0
healthy volunteers	1280	0

healthy volunteers	20	40
healthy volunteers	160	20
healthy volunteers	1280	0
healthy volunteers	10	10
healthy volunteers	2560	0
healthy volunteers	640	0
healthy volunteers	10	0
healthy volunteers	80	0
healthy volunteers	10	0
healthy volunteers	320	40
healthy volunteers	10	0
healthy volunteers	10	0
healthy volunteers	10	40
healthy volunteers	10	40
healthy volunteers	10	0
healthy volunteers	20	0
healthy volunteers	10	0
healthy volunteers	640	0
healthy volunteers	640	0
healthy volunteers	320	0
healthy volunteers	1280	0
healthy volunteers	40	0

healthy volunteers	1280	0
healthy volunteers	0	0
healthy volunteers	320	0
healthy volunteers	160	20
healthy volunteers	320	0
healthy volunteers	640	0
healthy volunteers	160	40
healthy volunteers	640	0
healthy volunteers	640	0
healthy volunteers	640	40
healthy volunteers	2560	0
healthy volunteers	2560	0
healthy volunteers	1280	0
healthy volunteers	1280	40
healthy volunteers	640	0
healthy volunteers	320	20
healthy volunteers	640	0
healthy volunteers	2560	0
healthy volunteers	320	10
healthy volunteers	320	0
healthy volunteers	80	20
healthy volunteers	10	0

healthy volunteers	10	40
healthy volunteers	10	0
healthy volunteers	160	0
healthy volunteers	640	80
healthy volunteers	640	0
healthy volunteers	2560	0
healthy volunteers	2560	0
healthy volunteers	640	0
healthy volunteers	1280	0
healthy volunteers	1280	40
healthy volunteers	640	40
healthy volunteers	10	0
healthy volunteers	640	0
healthy volunteers	640	20
healthy volunteers	160	0
healthy volunteers	10	20
healthy volunteers	160	0
healthy volunteers	10	160
healthy volunteers	10	0
healthy volunteers	0	0
healthy volunteers	320	0
healthy volunteers	80	20

healthy volunteers	1280	0
healthy volunteers	80	0
healthy volunteers	2560	40
healthy volunteers	10	0
healthy volunteers	640	40
healthy volunteers	10	0
healthy volunteers	10	0
healthy volunteers	320	0
healthy volunteers	10	0
healthy volunteers	10	0
healthy volunteers	10	0
healthy volunteers	640	0
healthy volunteers	2560	40
healthy volunteers	1280	0
healthy volunteers	5120	0
healthy volunteers	10	0
healthy volunteers	80	0
healthy volunteers	10	0
healthy volunteers	160	0
healthy volunteers	320	0
healthy volunteers	20	0
healthy volunteers	640	0

healthy volunteers	10	0
healthy volunteers	2560	0
healthy volunteers	1280	0
healthy volunteers	2560	0
healthy volunteers	1280	0
healthy volunteers	320	80
healthy volunteers	640	0
healthy volunteers	2560	0
healthy volunteers	640	0
healthy volunteers	320	0
healthy volunteers	80	20
healthy volunteers	2560	0
healthy volunteers	20	20
healthy volunteers	80	0
healthy volunteers	160	40
healthy volunteers	40	0
healthy volunteers	2560	0
healthy volunteers	40	0
healthy volunteers	-	0
healthy volunteers	640	0
healthy volunteers	1280	0
healthy volunteers	160	0

healthy volunteers	40	80
healthy volunteers	40	0
healthy volunteers	10	160
healthy volunteers	640	20
healthy volunteers	640	0
healthy volunteers	1280	0
healthy volunteers	10	0
healthy volunteers	640	0
healthy volunteers	640	0
healthy volunteers	1280	0
healthy volunteers	80	0
healthy volunteers	160	0
healthy volunteers	640	0
healthy volunteers	640	0
healthy volunteers	640	0
healthy volunteers	80	40
healthy volunteers	320	0
healthy volunteers	10	0
healthy volunteers	40	0
healthy volunteers	10	0
healthy volunteers	10	0
healthy volunteers	1280	80

healthy volunteers	80	0
healthy volunteers	640	0
healthy volunteers	2560	10
healthy volunteers	10	0
healthy volunteers	1280	0
healthy volunteers	1280	40
healthy volunteers	0	0
healthy volunteers	10	80
healthy volunteers	1280	0
lung cancer	320	0
lung cancer	320	20
lung cancer	40	40
lung cancer	160	40
lung cancer	10	160
lung cancer	320	0
lung cancer	80	80
lung cancer	10	0
lung cancer	320	20
lung cancer	0	80
lung cancer	320	40
lung cancer	10	20
lung cancer	160	40

lung cancer	80	0
lung cancer	160	320
lung cancer	40	320
lung cancer	80	40
lung cancer	320	20
lung cancer	160	40
lung cancer	160	20
lung cancer	320	10
lung cancer	640	10
lung cancer	80	20
lung cancer	1280	20
lung cancer	80	20
lung cancer	80	10
lung cancer	640	10
lung cancer	80	10
lung cancer	80	20
lung cancer	320	10
lung cancer	10	10
lung cancer	20	10
lung cancer	10	10
lung cancer	80	20
lung cancer	160	10

lung cancer	80	40
lung cancer	160	20
lung cancer	10	40
lung cancer	160	10
lung cancer	320	20
lung cancer	640	40
lung cancer	320	0
lung cancer	1280	20
lung cancer	20	10
lung cancer	20	10
lung cancer	160	10
lung cancer	0	0
lung cancer	0	20
lung cancer	0	40
lung cancer	0	80
lung cancer	320	10
lung cancer	2560	160
lung cancer	160	80
lung cancer	160	0
lung cancer	10	0
lung cancer	160	80
lung cancer	80	40

lung cancer	2560	0
lung cancer	640	0
lung cancer	1280	160
lung cancer	10	0
lung cancer	80	160
lung cancer	10	40
lung cancer	80	10
lung cancer	80	20
lung cancer	160	10
lung cancer	10	10
lung cancer	20	10
lung cancer	320	20
lung cancer	10	10
lung cancer	10	10
lung cancer	20	10
lung cancer	10	10
lung cancer	80	10
lung cancer	40	20
lung cancer	320	10
lung cancer	20	20
lung cancer	320	10
lung cancer	10	10

lung cancer	10	40
laryngeal cancer	640	40
laryngeal cancer	640	40
laryngeal cancer	640	0
laryngeal cancer	160	0
laryngeal cancer	80	0
laryngeal cancer	160	0
laryngeal cancer	160	0
laryngeal cancer	320	0
laryngeal cancer	640	0
laryngeal cancer	320	0
laryngeal cancer	20	0
laryngeal cancer	640	0
laryngeal cancer	0	0
laryngeal cancer	320	0
laryngeal cancer	160	0
laryngeal cancer	160	0
laryngeal cancer	640	0
laryngeal cancer	640	40
laryngeal cancer	80	0
laryngeal cancer	10	0
laryngeal cancer	320	0

laryngeal cancer	80	0
laryngeal cancer	320	0
laryngeal cancer	80	0
laryngeal cancer	10	0
laryngeal cancer	10	40
laryngeal cancer	320	160
laryngeal cancer	320	160
laryngeal cancer	320	0
laryngeal cancer	160	0
laryngeal cancer	320	0
laryngeal cancer	40	160
laryngeal cancer	320	40
laryngeal cancer	160	0
laryngeal cancer	160	80
oropharyngeal cancer	1280	80
oropharyngeal cancer	10	40
oropharyngeal cancer	80	0
oropharyngeal cancer	0	0
oropharyngeal cancer	160	40
oropharyngeal cancer	160	0
oropharyngeal cancer	320	80
oropharyngeal cancer	160	80

oropharyngeal cancer	10	40
esophageal cancer	160	10
esophageal cancer	320	20
esophageal cancer	80	10
esophageal cancer	0	20
esophageal cancer	0	20
esophageal cancer	320	0
esophageal cancer	160	10
esophageal cancer	0	40
esophageal cancer	0	0
esophageal cancer	10	10
esophageal cancer	80	0
esophageal cancer	0	40
esophageal cancer	320	10
esophageal cancer	0	0
esophageal cancer	160	10
esophageal cancer	20	0
esophageal cancer	80	20
esophageal cancer	0	20
esophageal cancer	320	10
esophageal cancer	640	20
esophageal cancer	320	10

esophageal cancer	80	10
esophageal cancer	10	0
esophageal cancer	640	0
esophageal cancer	80	40
esophageal cancer	320	0
esophageal cancer	320	0
esophageal cancer	320	0
esophageal cancer	320	0
esophageal cancer	320	0
esophageal cancer	20	40
esophageal cancer	80	10
esophageal cancer	320	80
esophageal cancer	160	0
esophageal cancer	80	10
esophageal cancer	20	10
esophageal cancer	1280	10
esophageal cancer	320	0
esophageal cancer	640	20
esophageal cancer	80	0
esophageal cancer	320	0
esophageal cancer	10	10
esophageal cancer	1280	10

esophageal cancer	10	10
esophageal cancer	10	0
esophageal cancer	10	0
esophageal cancer	0	0
esophageal cancer	320	20
esophageal cancer	320	80
esophageal cancer	10	40
esophageal cancer	640	10
esophageal cancer	320	10
esophageal cancer	0	40
esophageal cancer	10	40
esophageal cancer	0	10
esophageal cancer	10	80
esophageal cancer	0	20
esophageal cancer	10	10
esophageal cancer	0	20
esophageal cancer	1280	10
esophageal cancer	160	20
esophageal cancer	160	0
esophageal cancer	0	10
esophageal cancer	160	10
esophageal cancer	0	10

esophageal cancer	640	0
esophageal cancer	320	20
esophageal cancer	1280	0
esophageal cancer	10	10
esophageal cancer	320	0
esophageal cancer	160	10
esophageal cancer	160	0
esophageal cancer	1280	40
esophageal cancer	1280	20
esophageal cancer	80	0
esophageal cancer	80	20
gastric cancer	80	20
gastric cancer	80	20
gastric cancer	160	20
gastric cancer	80	10
gastric cancer	40	10
gastric cancer	20	20
gastric cancer	160	10
gastric cancer	10	20
gastric cancer	160	20
gastric cancer	640	20
gastric cancer	80	10

gastric cancer	40	40
gastric cancer	320	20
gastric cancer	10	40
gastric cancer	20	20
gastric cancer	40	20
gastric cancer	160	20
gastric cancer	80	10
gastric cancer	40	20
gastric cancer	80	40
gastric cancer	160	10
gastric cancer	640	20
gastric cancer	640	20
gastric cancer	160	20
gastric cancer	10	10
gastric cancer	0	20
gastric cancer	10	20
gastric cancer	160	20
gastric cancer	80	20
gastric cancer	40	20
gastric cancer	10	10
gastric cancer	20	20
gastric cancer	40	10

gastric cancer	320	10
gastric cancer	10	20
gastric cancer	40	20
gastric cancer	10	40
gastric cancer	80	20
gastric cancer	320	40
gastric cancer	0	40
cervical cancer	0	20
cervical cancer	0	0
cervical cancer	0	160
cervical cancer	0	40
cervical cancer	40	0
cervical cancer	0	0
cervical cancer	0	0
cervical cancer	40	0
cervical cancer	640	0
cervical cancer	40	0
cervical cancer	160	20
cervical cancer	20	0
cervical cancer	0	80
cervical cancer	20	80
cervical cancer	-	0

cervical cancer	160	0
cervical cancer	320	0
cervical cancer	320	0
cervical cancer	0	80
cervical cancer	0	0
cervical cancer	10	0
cervical cancer	0	10
cervical cancer	0	0
cervical cancer	160	10
cervical cancer	0	80
cervical cancer	10	0
cervical cancer	10	0
cervical cancer	2560	0
cervical cancer	0	0
cervical cancer	0	0
cervical cancer	20	20
cervical cancer	10	40
cervical cancer	320	0
cervical cancer	1280	0
cervical cancer	1280	0
cervical cancer	10	80
cervical cancer	0	0

cervical cancer	0	0
cervical cancer	0	0
cervical cancer	40	0
cervical cancer	0	0
cervical cancer	160	40
cervical cancer	10	0
cervical cancer	0	10
cervical cancer	20	0
cervical cancer	0	0
cervical cancer	0	20
cervical cancer	320	10
cervical cancer	40	0
cervical cancer	80	0
cervical cancer	80	0
cervical cancer	0	0
cervical cancer	0	0
cervical cancer	320	40
cervical cancer	0	0
cervical cancer	320	0
cervical cancer	160	0
cervical cancer	160	0
cervical cancer	80	0

cervical cancer	20	0
cervical cancer	640	0
cervical cancer	1280	0
cervical cancer	320	0
cervical cancer	0	160
cervical cancer	160	0
cervical cancer	0	0
cervical cancer	10	0
cervical cancer	20	80
cervical cancer	20	0
cervical cancer	640	0
cervical cancer	80	0
cervical cancer	0	40
cervical cancer	0	0
cervical cancer	80	0
cervical cancer	2560	0
cervical cancer	10	0
cervical cancer	0	0
cervical cancer	160	0
cervical cancer	640	0
cervical cancer	320	10
cervical cancer	80	40

cervical cancer	10	0
cervical cancer	10	0
cervical cancer	0	0
cervical cancer	0	20
cervical cancer	0	20
cervical cancer	0	0
cervical cancer	80	10
cervical cancer	320	10
cervical cancer	80	160
cervical cancer	20	40
cervical cancer	80	
cervical cancer	160	0
cervical cancer	320	320
cervical cancer	20	10
cervical cancer	160	0
cervical cancer	80	10
cervical cancer	80	0
cervical cancer	10	0
cervical cancer	10	0
cervical cancer	10	160
cervical cancer	0	80
cervical cancer	20	80

cervical cancer	640	40
cervical cancer	1280	0
cervical cancer	320	0
cervical cancer	0	0
cervical cancer	0	0
cervical cancer	0	160
cervical cancer	20	0
cervical cancer	320	0
cervical cancer	10	40
cervical cancer	0	40
cervical cancer	640	0
cervical cancer	160	0
cervical cancer	0	0
cervical cancer	320	0
cervical cancer	320	0
cervical cancer	160	0
cervical cancer	10	40
colon cancer	320	80
colon cancer	40	160
colon cancer	640	0
colon cancer	320	160
colon cancer	160	160

colon cancer	0	160
colon cancer	0	80
colon cancer	320	320
colon cancer	80	40
colon cancer	40	0
colon cancer	40	160
colon cancer	0	80
colon cancer	0	80
colon cancer	0	40
colon cancer	20	80
colon cancer	80	80
colon cancer	2560	40
colon cancer	10	160
colon cancer	10	80
colon cancer	10	0
colon cancer	80	320
colon cancer	40	0
colon cancer	20	320
colon cancer	10	0
colon cancer	20	0
colon cancer	10	320
colon cancer	80	0

colon cancer	10	80
colon cancer	640	40
colon cancer	10	0
colon cancer	0	160
colon cancer	0	80
colon cancer	80	0
colon cancer	80	0
colon cancer	80	80
colon cancer	80	0
colon cancer	80	0
colon cancer	160	40
colon cancer	40	40
colon cancer	160	40
rectal cancer	0	80
rectal cancer	160	80
rectal cancer	40	0
rectal cancer	0	0
rectal cancer	160	0
rectal cancer	40	0
rectal cancer	1280	40
rectal cancer	320	80
rectal cancer	0	20

rectal cancer	10	40
rectal cancer	160	40
rectal cancer	320	0
rectal cancer	10	40
rectal cancer	160	0
rectal cancer	10	0
rectal cancer	320	20
rectal cancer	80	40
rectal cancer	10	0
rectal cancer	10	40
rectal cancer	20	80
rectal cancer	20	0
rectal cancer	10	0
rectal cancer	320	0
rectal cancer	320	40
rectal cancer	160	0
rectal cancer	80	80
rectal cancer	320	40
rectal cancer	10	0
rectal cancer	0	0
rectal cancer	160	20
rectal cancer	10	320

rectal cancer	160	0
rectal cancer	20	0
rectal cancer	40	40
rectal cancer	10	0
rectal cancer	-	40
rectal cancer	0	0
rectal cancer	10	0
rectal cancer	20	20
rectal cancer	160	0
